# Collaboration Between Nursing, Psycology and Psychiatry in an Intensive Outpatient Program for Suicidal Ideation

**DOI:** 10.1192/j.eurpsy.2025.2364

**Published:** 2025-08-26

**Authors:** T. Bollain Muñoz, M. Valtueña Garcia, J. Sanchez Adsuara, M. L. Barrigón Estevez, R. Crespo Rubio, I. Oliveira Amat

**Affiliations:** 1 HGUGM, Madrid, Spain

## Abstract

**Introduction:**

The collaboration between nursing, psychology, and psychiatry is essential for delivering comprehensive and effective mental health care. A multidisciplinary approach ensures that patients receive holistic support where everyone is aligned on the patient’s needs, treatment progress, and level of risk, and drawing on the unique skills and expertise of each discipline. Without proper coordination, there is a higher risk of gaps in care, conflicting interventions, or misunderstandings that could negatively impact the patient’s safety and well-being.

**Objectives:**

To highlight the importance of collaborative work between nursing, psychology, and psychiatry within the context of an acute, short-term, intensive outpatient program for suicidal ideation, such as PRISURE, is essential. For patients at high risk of suicide, particularly those experiencing acute symptoms, a combined multidisciplinary approach is critical to providing effective and timely care.

**Methods:**

The program distinguishes between two types of interventions: an intensive program and a regular program, both offering a couple months long intervention but differing in the frequency of visits. The entire team meets weekly to assess new cases and discuss patients within the intensive program. An additional meeting is held to coordinate care for patients in the regular program between nursing and psychiatry. Regular multidisciplinary meetings are key to ensuring a coherent and unified approach across both programs. Appointment schedules are carefully coordinated to minimize the time between consultations, ensuring continuous and consistent follow-up for patients. The program also coordinates with regular mental health out-patient clinics within the public health system, to garantee a good transition of care.

**Results:**

During these collaborative meetings, each specialist shared their assessments and observations on the patient’s progress, enabling the team to develop a unified therapeutic plan. Any changes in symptoms or new events are promptly communicated among all treating professionals, allowing for a rapid and coordinated multidisciplinary response. The diverse perspectives of each team member contribute to a more nuanced and comprehensive understanding of the patient’s needs and treatment.

**Conclusions:**

In summary, the collaboration between nursing, psicology and psychiatry creates a synergistic approach that is essential for delivering high-quality, patient-centered mental health care, particularly for those experiencing suicidal ideation.

**Disclosure of Interest:**

None Declared

